# One Week Hypofractionated Adjuvant Radiation for Early Breast Cancer Patients Treated at a Tertiary Cancer Centre in South India: A Comparative Dosimetric Study of Forward Intensity-Modulated Radiotherapy (F-IMRT) and Volumetric Modulated Arc Therapy (VMAT)

**DOI:** 10.1155/ijbc/4267362

**Published:** 2025-06-28

**Authors:** Ashwini Gopal, M. L. Prem Kumar, Prathusha Chitrala, Heena Kauser, A. Krishnam Raju, V. Sudhakar Kumar, Srilatha Cheera, P. V. Arun, N. V. N. M. Sresty, G. Deleep Kumar

**Affiliations:** ^1^Department of Radiation Oncology, Basvatarakam Indo American Cancer Hospital and Research Institute, Hyderabad, India; ^2^Department of Medical Physics, Basvatarakam Indo American Cancer Hospital and Research Institute, Hyderabad, India

**Keywords:** forward intensity-modulated radiotherapy and volumetric modulated arc therapy, left breast cancer, oar doses

## Abstract

**Objective:** This study is aimed at comparing the forward IMRT (F-IMRT) and VMAT techniques in the adjuvant treatment of left-sided breast cancer using hypofractionated radiation over 1 week with deep inspiration breath hold (DIBH) via the Elekta Active Breathing Coordinator (ABC) system.

**Materials and Methods:** Treatment plans for 26 patients receiving 26 Gy in five fractions (5.2 Gy/fraction), followed by a 10-Gy electron boost in five fractions, were analyzed. The boost phase was excluded from the comparison. F-IMRT and VMAT plans were evaluated for dose to 95% of the volume, conformity index (CI), mean left lung dose (MLLD), left lung V8 Gy, mean heart dose (MHD), heart V1.5Gy and V7Gy, mean right breast dose (MRBD), and mean right lung dose (MRLD). Statistical analysis was conducted using the Wilcoxon signed-rank test.

**Results:** PTV coverage was similar in F-IMRT and VMAT arms (95.83% vs. 95.38%), but CI was significantly improved with VMAT (1.31 vs. 1.04). F-IMRT significantly reduced MLLD (4.55 Gy vs. 5.95 Gy) and left lung V8Gy (18.78% vs. 25.87%) when compared to VMAT. MHD was lower with F-IMRT (1.79Gy vs. 2.47Gy), with significantly reduced heart V1.5Gy (21.6% vs. 54.4%) when compared to VMAT, with V7Gy not different (5.04% vs. 5.79%) between F-IMRT and VMAT. F-IMRT also resulted in lower MRBD (0.62 Gy vs. 2.4 Gy) and MRLD (0.38 Gy vs. 1.8 Gy) when compared to VMAT.

**Conclusion:** F-IMRT provides comparable target coverage to VMAT while significantly reducing radiation exposure to the heart, lungs, and contralateral breast for left-sided breast cancer treatment with DIBH and hypofractionation over 1 week. Given its dosimetric advantages, F-IMRT should be the preferred technique to enhance patient safety and minimize long-term toxicities.

## 1. Introduction

Hypofractionated radiotherapy over 1 week has been used in the adjuvant treatment of early breast cancer since the results of the Fast-Forward trial. The 5-year follow-up of the study showed no significant difference in ipsilateral breast tumor recurrences (IBTRs) and late normal tissue side effects in the breast when compared to standard hypofractionated radiotherapy (40.05 Gy in 15 fractions). The incidence of radiation-induced heart disease and symptomatic lung fibrosis was low [1].

According to the EBCTCG, the incidence of contralateral breast cancer and heart disease increased after 15 years. Heart disease is the most common cause of nonbreast cancer mortality in breast cancer patients who have undergone radiation, with a risk ratio of 1.27. It is likely that older radiation techniques could be responsible for late toxicities such as heart disease. With the advent of newer technologies and techniques, such as deep inspiration breath hold (DIBH) and image guidance, we expect reduced toxicities, not only acute but also long-term late effects. [2]

Radiation therapy has evolved from conventional fractionation (2 Gy per fraction) to hypofractionated treatment, with a dose per fraction of 2.67 Gy as the standard of care after four randomized trials published long-term data such as START-A, START-B, RMH/GOC, and Ontario Clinical Oncology Group (OCOG) with similar toxicities and IBTRs [3–6]. With the Fast-Forward regimen, where the dose per fraction was 5.2 Gy and with higher doses per fraction, we naturally expect to see an increase in acute and late toxicities such as skin reactions, breast fibrosis, and breast shrinkage. The dose per fraction significantly impacts both acute and late toxicities [7, 8].

Conventionally, tangential fields in radiation were used to treat breast cancer. Currently, with the advent of newer radiation techniques such as DIBH and image guidance for planning and delivery, the aim is to reduce normal tissue dose and improve treatment precision. This study evaluates the dosimetry between F-IMRT and volumetric modulated arc therapy (VMAT) in treating left-sided breast cancer, with the aim of determining the most suitable technique for whole breast irradiation (WBI) using the Fast-Forward protocol delivered in 1 week.

## 2. Materials and Methods

Twenty-six patients with early stage left-sided breast cancer eligible for WBI were included in the study and were treated with Fast-Forward protocol using DIBH and image guidance at our institute between September 2022 and August 2024. Patients who received supraclavicular radiation and axillary radiation were excluded.

The research protocol was reviewed and approved by the Institutional Ethics Committee, and all patient data were anonymized.

### 2.1. Radiation Treatment Planning

Patients were immobilized in the supine position using a Vac-Lok system. CT simulation was performed with a 2.5-mm slice thickness, capturing two scans: one in free breathing and the other in DIBH using the Elekta Active Breathing Coordinator (ABC) system.

The clinical target volume (CTV) and planning target volume (PTV) were contoured following the Fast-Forward protocol. The prescribed dose was 26 Gy in five fractions (5.2 Gy per fraction) over 1 week in five daily fractions were deliver and followed with a 10 Gy electron boost in five fractions for all patients. The boost phase was excluded from the evaluation.

### 2.2. Planning Parameters as per Fast Forward Trial

Ninety-five percent PTV coverage is required to receive 95% dose. Organ-at-risk (OAR) dose constraints are as follows:

• Ipsilateral lung: V8 Gy < 15%
• Heart: V1.5 Gy < 30%, V7 Gy < 5%

### 2.3. Treatment Planning

Two plans were generated for each patient using the Monaco treatment planning system Version 6.1.2.0 (Elekta, Sweden):

1. Forward IMRT (F-IMRT): 2–4 beams, field-in-field technique, predominantly half-beam tangential
2. VMAT: single half arc or two half arcs
3. Dosimetry parameters evaluated:
  • Dose to 95% of PTV (D95%)
  • Conformity index (CI)
  • Mean left lung dose (MLLD)
  • Left lung—V_8 Gy_
  • Mean right lung dose (MRLD)
  • Mean heart dose (MHD)
  • Heart—V_1.5 Gy_ and V_7 Gy_
  • Mean right breast dose (MRBD)

### 2.4. Statistical Analysis

Data were compiled into an Excel sheet and analyzed using the Wilcoxon matched-pair signed-rank test. A *p* value < 0.05 was considered statistically significant.

## 3. Results

Twenty-six patients with early stage left-sided breast cancer eligible for WBI were analyzed, and all patients were treated with DIBH with image guidance using the Elekta ABC system, with the breathing threshold varying from 1 to 1.3 L. All F-IMRT and VMAT plans for these 26 patients were analyzed by treating radiation oncologists.

The dosimetric parameters are summarized in Table 1.

### 3.1. Target Volume Dosimetry

• D95%—Dose received by 95% PTV was comparable between F-IMRT and VMAT (95.83% vs. 95.38%, *p* = 0.68).
• The CI was significantly higher in F-IMRT (1.31 vs. 1.04, *p* < 0.00001) when compared to VMAT.

### 3.2. OAR Dosimetry

#### 3.2.1. Ipsilateral Lung

• The MLLD was lower in F-IMRT (4.55 Gy vs. 5.95 Gy, *p* < 0.00022).
• The volume receiving 8 Gy (V8 Gy) was also lesser in F-IMRT (18.78% vs. 25.87%, *p* = 0.00014) when compared to VMAT.

#### 3.2.2. Contralateral Lung

• The MRLD was significantly lower with F-IMRT (0.38 Gy vs. 1.8 Gy, *p* < 0.0001) when compared to VMAT.

### 3.3. Heart

• The MHD was lower in F-IMRT (1.79 Gy vs. 2.47 Gy, *p* = 0.00168) when compared to VMAT.
• The volume receiving 1.5 Gy (V1.5 Gy) was significantly lower in F-IMRT (21.6% vs. 54.4%, *p* < 0.00001) when compared to VMAT.
• The volume receiving 7 Gy (V7 Gy) showed no significant difference (5.04% vs. 5.79%, *p* = 0.40) between F-IMRT and VMAT.

### 3.4. Contralateral Breast

• The MRBD was lower in F-IMRT (0.62 Gy vs. 2.4 Gy, p = 0.00001) when compared to VMAT.

### 3.5. Summary

The dose received by 95% of the PTV volume was similar between F-IMRT and VMAT, but in terms of CI, VMAT plans were more conformal when compared to F-IMRT. MLLD, MRLD, MHD, and MRBD were lesser in F-IMRT plans when compared to VMAT plans. Volume doses for left lung (V8 Gy) and heart (V1.5 Gy) were lesser in F-IMRT plans when compared to VMAT plans. Volume doses for heart (V7 Gy) were similar in both F-IMRT and VMAT plans.

**Figure 1:**
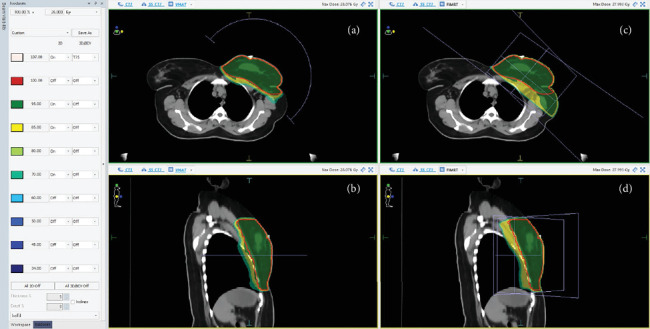
Dose distribution in a representative patient. (a, b) VMAT plan; (c, d) F-IMRT plan.

**Figure 2:**
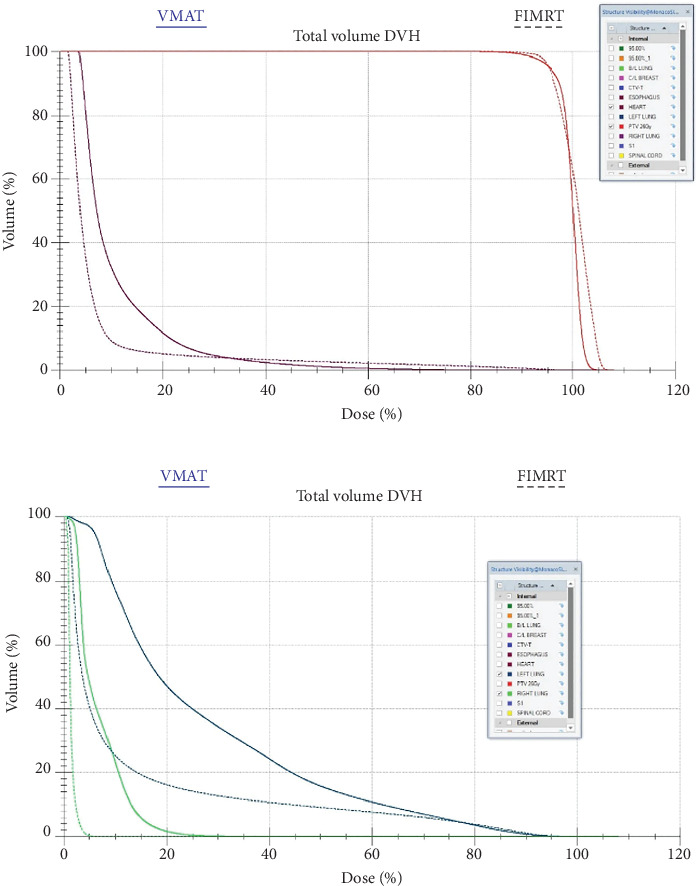
Dose–volume histogram (DVH) comparison between VMAT and F-IMRT in a representative patient. (a) Heart dose with planning target volume (PTV) coverage. (b) Right lung (light green) and left lung (dark green) dose comparison.

Figure 1 shows the dose distribution in a representative patient for both VMAT and F-IMRT techniques. As depicted in Figure 2, the dose–volume histograms (DVHs) highlight the dosimetric advantages of F-IMRT over VMAT in terms of heart and lung dose reduction.

## 4. Discussion

Radiation improves local control and survival in early breast cancer patients who undergo breast conservation surgery. However, it is also associated with late toxicities such as second malignancies and heart disease. Among nonbreast cancer–related mortalities, cardiac diseases, and lung cancer are the most common causes [2].

Historically, the conventional fractionated regimen of 50 Gy in 25 fractions (2 Gy per fraction over 5 weeks) was used for breast adjuvant radiation (NSABP) [3]. Breast adenocarcinomas are sensitive to fraction size, and an *α*/*β* value of around 3 has been estimated [7]. This led to the beginning of hypofractionation in breast cancer treatment. With evidence from the UK START trials and the Canadian RMH/GOC and OCOG study, hypofractionated radiation (40.05 Gy in 15 fractions, 2.67 Gy per fraction) has become standard of care for many years, as it has similar efficacy and reduced normal tissue reactions compared to conventional radiotherapy [4–6].

The Fast-Forward trial further reduced the duration of radiation therapy to 1 week, administering 26 Gy in five fractions (5.2 Gy per fraction) in patients without regional lymph node irradiation. The 5-year results on ipsilateral breast cancer recurrence and normal tissue effects showed a marked reduction compared to standard hypofractionation. [1] Complications such as rib fractures, pericarditis, tissue necrosis, and sarcoma have been documented, though the incidence is low (< 5%) with median follow-up of 6 years in these studies [9]. Additionally, major coronary events increase by 7.5/Gy following radiation, and this rate increases further in patients with pre-existing cardiac conditions. The increase in ischemic heart disease begins a few years after radiation and continues for up to 20 years [10].

The evolution of radiation therapy for breast cancer started with conventional 2D techniques using X-ray simulators with bony landmarks and has advanced to more conformal techniques using computed tomography, such as 3D conformal radiation (3DCRT), and currently, intensity-modulated radiation therapy (IMRT) and VMAT. It is recommended to use a radiotherapy technique that minimizes dose to the heart and lungs [11]. With increasing survival rates in breast cancer patients due to advances in surgical, medical, and radiation therapies, it is crucial to treat precisely and reduce both acute and late side effects.

Several studies have evaluated optimal techniques for left breast irradiation. One study comparing IMRT and VMAT in patients who underwent mastectomy or breast conservation surgery found that IMRT resulted in superior OAR dose reduction compared to VMAT while maintaining comparable target volume coverage. The prescribed dose was 50.4 Gy in 28 fractions, including irradiation of the breast or chest wall and regional nodes (axilla and supraclavicular lymph nodes) [12]. However, in 24 left breast cancer patients who underwent postmastectomy, patients benefited from VMAT when compared to IMRT with regards to reduced doses to lung and heart [13]. Another dosimetric comparison involving 12 breast cancer patients concluded that VMAT was not superior to IMRT or conventional radiotherapy [14]. Similarly, in a study of 35 left breast cancer patients after postmastectomy, inverse planning demonstrated increased target coverage with VMAT while reducing high-dose OAR exposure. However, low-dose OAR exposure was higher with VMAT compared to 3DCRT and IMRT [15]. Additionally, VMAT plans have shown a reduction in high-dose exposure to the heart and lung but an increase in low-dose exposure, as observed in 30 patients treated with hypofractionated radiation to the breast [16]. Another dosimetric comparison of 21 patients receiving 1-week hypofractionated radiation found that both 3DCRT and VMAT were feasible, with VMAT increasing the volume receiving 5 Gy to the heart, whereas 3DCRT increased the ipsilateral lung and skin dose [17].

In our study, we compared F-IMRT and VMAT plans for left breast cancer patients who underwent breast conservation surgery and received adjuvant radiation to breast without lymph nodal irradiation with hypofractionation by Fast-Forward protocol with DIBH and image guidance. Our findings revealed that F-IMRT resulted in significantly lower mean lung doses (both ipsilateral and contralateral) and reduced V8 Gy exposure to the ipsilateral lung compared to VMAT. Furthermore, the MHD and V1.5 Gy to the heart were significantly lower in F-IMRT plans, while V7 Gy to the heart showed no significant difference between the two techniques. Additionally, the mean dose to the contralateral breast was significantly lower in F-IMRT plans.

Although both techniques achieved similar target volume coverage at 95% of the prescribed dose (V95–95%), VMAT plans demonstrated better dose conformality. However, given the long-term risks of cardiovascular disease and second malignancies, which increase over 15–20 years postadjuvant radiation therapy, minimizing radiation exposure to OARs is critical. Due to the limited data on ultra-hypofractionated 1-week regimens, we emphasize the importance of reducing doses to the heart, lung, and contralateral breast using all available techniques. This includes employing DIBH for eligible patients and prioritizing F-IMRT to minimize OAR doses. Additionally, image guidance should be used to ensure accurate radiation delivery.

Despite the limitations of a small sample size and retrospective data, our findings support the use of F-IMRT in all breast cancer patients requiring ultra-hypofractionated 1-week radiation therapy to the left breast only, without nodal irradiation.

F-IMRT plans are superior to VMAT plans in left breast cancer patients undergoing ultra-hypofractionated 1-week adjuvant radiation therapy following breast conservation surgery. F-IMRT significantly reduces OAR doses compared to VMAT. Therefore, we recommend F-IMRT for all left-breast-only radiotherapy patients, utilizing DIBH and precise image guidance to optimize treatment outcomes.

## 5. Conclusion

F-IMRT plans were superior to VMAT plans in left breast cancer patients undergoing ultra-hypofractionated 1-week adjuvant radiation therapy following breast conservation surgery. F-IMRT significantly reduces OAR doses compared to VMAT. Therefore, we recommend F-IMRT for all left-breast-only radiotherapy patients, utilizing DIBH and precise image guidance to optimize treatment outcomes.

## Figures and Tables

**Table 1:** Comparison of dosimetric parameters of PTV and OAR.

	**FIMRT**	**VMAT**	**p** ** value**
Dose received by 95% volume of PTV (%)	95.83	95.38	0.68
CI	1.31	1.04	< 0.00001
MLLD (Gy)	4.55	5.95	< 0.00022
V8 left lung (%)	18.78	25.87	0.00014
Mean right lung dose (Gy)	0.38	1.8	< 0.0001
MHD (Gy)	1.79	2.47	0.00168
V1.5 Gy H (%)	21.6	54.4	< 0.00001
V7 Gy H (%)	5.04	5.79	0.40
MRB (Gy)	0.62	2.4	0.00001

## Data Availability

The data that support the findings of this study are available from the corresponding author upon reasonable request.

## References

[1] Murray Brunt A., Haviland J. S., Wheatley D. A. (2020). Hypofractionated Breast Radiotherapy for 1 Week Versus 3 Weeks (FAST-Forward): 5-Year Efficacy and Late Normal Tissue Effects Results From a Multicentre, Non-Inferiority, Randomised, Phase 3 Trial. *Lancet*.

[2] Clarke M., Collins R., Darby S. (2005). Effects of Radiotherapy and of Differences in the Extent of Surgery for Early Breast Cancer on Local Recurrence and 15-Year Survival: An Overview of the Randomised Trials. *Lancet*.

[3] Fisher B., Anderson S., Bryant J. (2002). Twenty-Year Follow-Up of a Randomized Trial Comparing Total Mastectomy, Lumpectomy, and Lumpectomy Plus Irradiation for the Treatment of Invasive Breast Cancer. *The New England Journal of Medicine*.

[4] Haviland J. S., Owen J. R., Dewar J. A. (2013). The UK Standardisation of Breast Radiotherapy (START) Trials of Radiotherapy Hypofractionation for Treatment of Early Breast Cancer: 10-Year Follow-Up Results of Two Randomised Controlled Trials. *Lancet Oncology*.

[5] The START Trialists’ Group (2008). The UK Standardisation of Breast Radiotherapy (START) Trial B of Radiotherapy Hypofractionation for Treatment of Early Breast Cancer: A Randomised Trial. *Lancet*.

[6] The START Trialists’ Group (2008). The UK Standardisation of Breast Radiotherapy (START) Trial A of Radiotherapy Hypofractionation for Treatment of Early Breast Cancer: A Randomised Trial. *Lancet Oncology*.

[7] Yarnold J., Ashton A., Bliss J. (2005). Fractionation Sensitivity and Dose Response of Late Adverse Effects in the Breast After Radiotherapy for Early Breast Cancer: Long-Term Results of a Randomised Trial. *Radiotherapy and Oncology*.

[8] Fowler J. F. (1989). The Linear-Quadratic Formula and Progress in Fractionated Radiotherapy. *The British Journal of Radiology*.

[9] Pierce S. M., Recht A., Lingos T. I. (1992). Long-Term Radiation Complications Following Conservative Surgery (CS) and Radiation Therapy (RT) in Patients With Early Stage Breast Cancer. *International Journal of Radiation Oncology • Biology • Physics*.

[10] Darby S. C., Ewertz M., McGale P. (2013). Risk of Ischemic Heart Disease in Women After Radiotherapy for Breast Cancer. *The New England Journal of Medicine*.

[11] National Institute for Health and Care Excellence (2018). *Early and Locally Advanced Breast Cancer: Diagnosis and Management*.

[12] Negi P., Sellapandiyan R., Srivastava H., Anandhan G., Pruthi H. S., Singh P. (2023). Intensity-Modulated Radiation Therapy (IMRT) Versus Rapidarc in the Treatment of Carcinoma Left Breast–Finding the Optimal Radiation Therapy Technique. *Iranian Journal of Medical Physics*.

[13] Wang R., Shen J., Yan H. (2022). Dosimetric Comparison Between Intensity-Modulated Radiotherapy and Volumetric-Modulated Arc Therapy in Patients of Left-Sided Breast Cancer Treated With Modified Radical Mastectomy: CONSORT. *Medicine (Baltimore)*.

[14] Badakhshi H., Kaul D., Nadobny J., Wille B., Sehouli J., Budach V. (2013). Image-Guided Volumetric Modulated Arc Therapy for Breast Cancer: A Feasibility Study and Plan Comparison With Three-Dimensional Conformal and Intensity-Modulated Radiotherapy. *British Journal of Radiology*.

[15] Das Majumdar S. K., Amritt A., Dhar S. S. (2022). A Dosimetric Study Comparing 3D-CRT vs. IMRT vs. VMAT in Left-Sided Breast Cancer Patients After Mastectomy at a Tertiary Care Centre in Eastern India. *Cureus*.

[16] Prasun P., Kharade V., Pal V., Gupta M., Das S., Pasricha R. (2023). Dosimetric Comparison of Hypofractionated Regimen in Breast Cancer Using Two Different Techniques: Intensity-Modulated Radiation Therapy (IMRT) and Volumetric-Modulated Arc Therapy (VMAT). *Cureus*.

[17] Piras A., Menna S., D’Aviero A. (2022). New Fractionations in Breast Cancer: A Dosimetric Study of 3D-CRT Versus VMAT. *Journal of Medical Radiation Sciences*.

